# Supplementary material for: Seminoma and Embryonal Carcinoma Footprints Identified by Analysis of Integrated Genome-Wide Epigenetic and Expression Profiles of Germ Cell Cancer Cell Lines
Source: PLoS One. 2014 Jun 2;9(6):e98330. doi: 10.1371/journal.pone.0098330 (PMC4041891; doi:10.1371/journal.pone.0098330)
Supplement: File S1 — ZIP file containing DMRforPairs output for significant regions. Please start from the html files. (ZIP) [file pone.0098330.s008.zip › figures/13241.pdf]

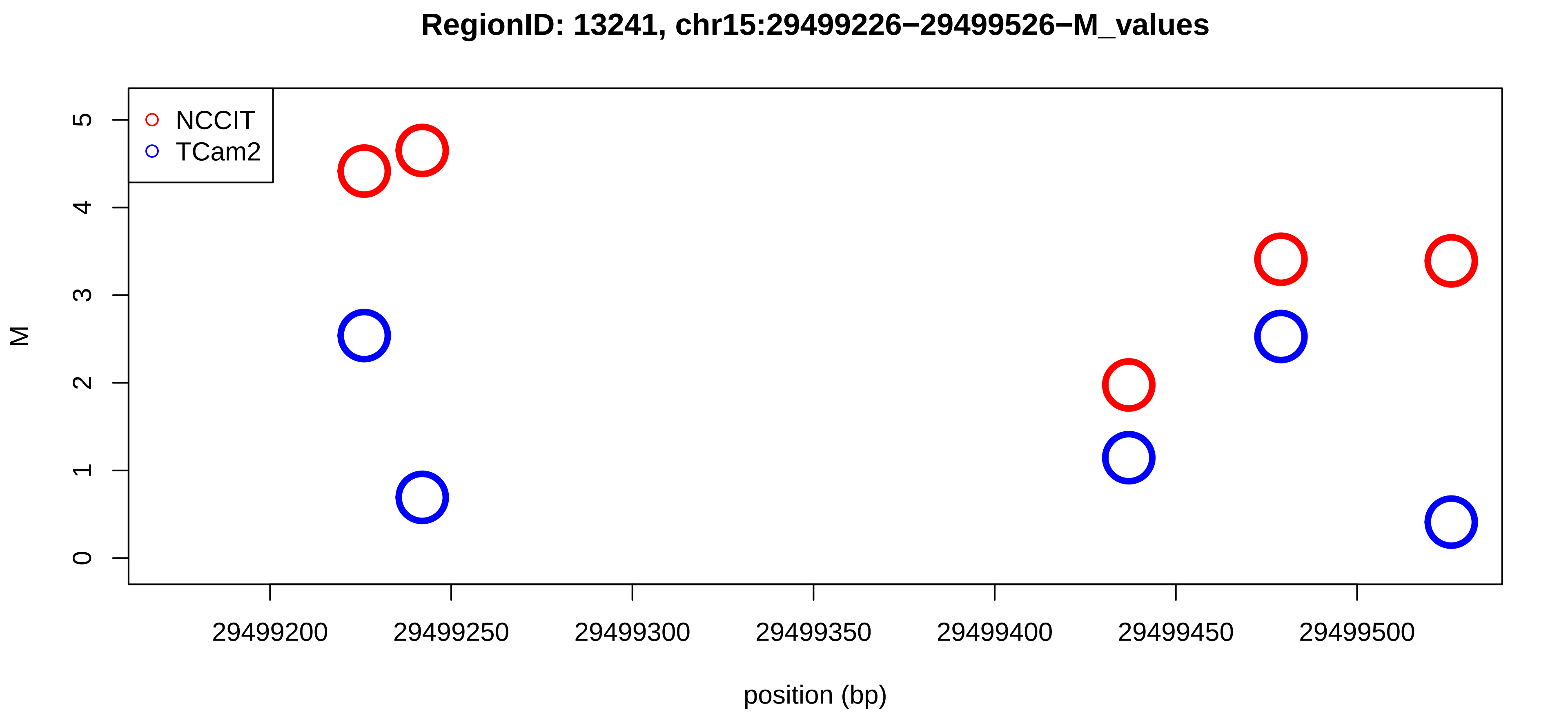
RegionID: 13241, chr15:29499226−29499526−M_values
NCCIT
TCam2
M
0
1
2
3
4
5
29499200
29499250
29499300
29499350
29499400
29499450
29499500
position (bp)

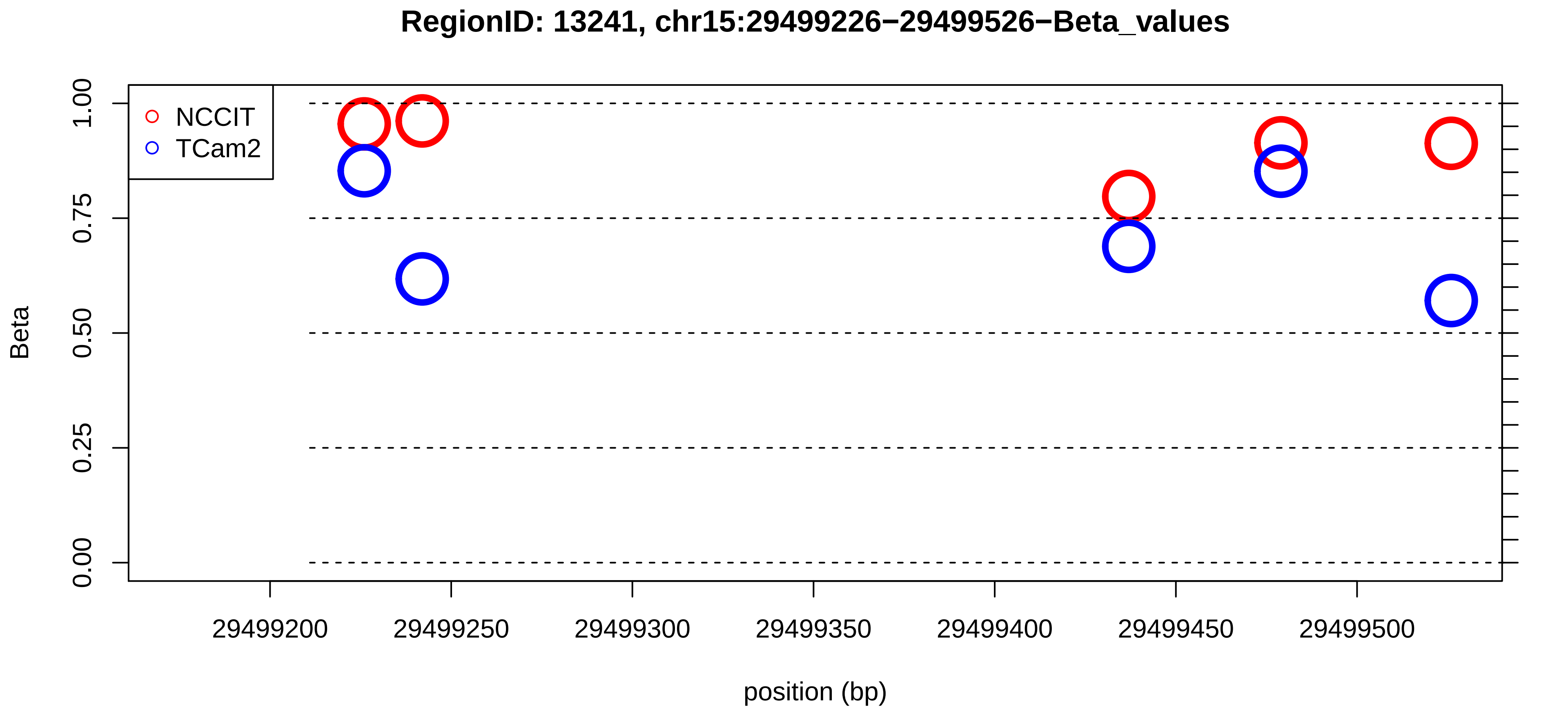
RegionID: 13241, chr15:29499226−29499526−Beta_values
NCCIT
TCam2
Beta
0.00
0.25
0.50
0.75
1.00
29499200
29499250
29499300
29499350
29499400
29499450
29499500
position (bp)
